# Study protocol for a multicenter randomized controlled trial to compare the efficacy of end-ischemic dual hypothermic oxygenated machine perfusion with static cold storage in preventing non-anastomotic biliary strictures after transplantation of liver grafts donated after circulatory death: DHOPE-DCD trial

**DOI:** 10.1186/s12876-019-0956-6

**Published:** 2019-03-12

**Authors:** Rianne van Rijn, Aad P. van den Berg, Joris I. Erdmann, Nigel Heaton, Bart van Hoek, Jeroen de Jonge, Henri G. D. Leuvenink, Shekar V. K. Mahesh, Sarah Mertens, Diethard Monbaliu, Paolo Muiesan, M. Thamara P. R. Perera, Wojciech G. Polak, Xavier Rogiers, Roberto I. Troisi, Yvonne de Vries, Robert J. Porte

**Affiliations:** 1 Section Hepatobiliary Surgery and Liver Transplantation, Department of Surgery, University Medical Center Groningen, Groningen, The Netherlands; 2 Surgical Research Laboratory, Department of Surgery, University Medical Center Groningen, Groningen, The Netherlands; 3 Department of Gastroenterology and Hepatology, University Medical Center Groningen, Groningen, The Netherlands; 4 Department of Surgery, Leiden University Medical Center, Leiden, The Netherlands; 5 Institute of Liver Studies, Kings College Hospital NHS Foundation Trust, London, UK; 6 Department of Gastroenterology and Hepatology, Leiden University Medical Center, Leiden, The Netherlands; 7 Department of Surgery, Erasmus University Medical Center, Rotterdam, The Netherlands; 8 Department of Radiology, University Medical Center Groningen, Groningen, The Netherlands; 9 Department of Abdominal Transplantation Surgery, University Hospitals of Leuven, Leuven, Belgium; 10 Liver Unit, Queen Elizabeth Hospital Birmingham, University Hospitals Birmingham NHS Foundation Trust, Birmingham, UK; 11 Department of Transplant Surgery, Ghent University Hospital, Ghent, Belgium

**Keywords:** Donation after circulatory death, Adult, Incidence, Ischemic type biliary lesions, Survival, Cost

## Abstract

**Background:**

The major concern in liver transplantation of grafts from donation after circulatory death (DCD) donors remains the high incidence of non-anastomotic biliary strictures (NAS). Machine perfusion has been proposed as an alternative strategy for organ preservation which reduces ischemia-reperfusion injury (IRI). Experimental studies have shown that dual hypothermic oxygenated machine perfusion (DHOPE) is associated with less IRI, improved hepatocellular function, and better preserved mitochondrial and endothelial function compared to conventional static cold storage (SCS). Moreover, DHOPE was safely applied with promising results in a recently performed phase-1 study. The aim of the current study is to determine the efficacy of DHOPE in reducing the incidence of NAS after DCD liver transplantation.

**Methods:**

This is an international multicenter randomized controlled trial. Adult patients (≥18 yrs. old) undergoing transplantation of a DCD donor liver (Maastricht category III) will be randomized between the intervention and control group. In the intervention group, livers will be subjected to two hours of end-ischemic DHOPE after SCS and before implantation. In the control group, livers will be subjected to care as usual with conventional SCS only. Primary outcome is the incidence of symptomatic NAS diagnosed by a blinded adjudication committee. In all patients, magnetic resonance cholangiography will be obtained at six months after transplantation.

**Discussion:**

DHOPE is associated with reduced IRI of the bile ducts. Whether reduced IRI of the bile ducts leads to lower incidence of NAS after DCD liver transplantation can only be examined in a randomized controlled trial.

**Trial registration:**

The trial was registered in Clinicaltrials.gov in September 2015 with the identifier NCT02584283.

## Background

Limited organ availability for liver transplantation remains a major concern [1]. Utilization of livers with suboptimal quality or so called “extended criteria” donors, such as older donors, donors with fatty livers and donation after circulatory death (DCD) donors, have reduced the organ deficit in recent years. In fact, the percentage of DCD donors in the USA has increased from 1.1% in 1995 to 11.2% in 2010 [2]. In Europe, the percentage of DCD in liver transplantation was as high as 35% in the Netherlands and 22% in the UK in 2015 [3, 4]. The poor post-transplant outcomes of these grafts have concurrently limited the utilization of these livers. The percentage of unused grafts increased from 9% in 2004 to 28% in 2010 in the USA which is mainly attributed to the growing number of DCD donors [2].

Non-anastomotic biliary strictures (NAS) are a major complication after liver transplantation and occur in 29% of patients receiving a DCD donor graft, compared to 11% among recipients of donation after brain death (DBD) liver grafts in the University Medical Center Groningen (unpublished data). Moreover, longer hospital stay and increased cost have been associated with NAS in DCD liver transplantation [5–7]. Among the variety of risk factors described to be associated with NAS, ischemia/reperfusion related injury is one of the most important factors. Donor warm ischemia and cold ischemia during static cold storage (SCS) have been associated with the development of NAS after DCD [6, 8]. Also, injury of the peribiliary vascular plexus and peribiliary glands is thought to play an important role in the development of NAS [9–11].

Due to the increased risk of complications, transplantation of DCD liver grafts necessitates the development of more qualified preservation methods than the conventional SCS. Machine perfusion (MP) is a dynamic preservation strategy which has been identified as a tool to improve the quality of DCD organs [12]. One of the most important benefits of MP compared to conventional SCS is the ability to provide oxygen to the graft. Even at very low temperatures such as 12 °C, liver metabolism still requires oxygen [13]. Other benefits of MP are the exposure of the endothelium to perfusion, the supply of nutrients, and dilution of waste products. Many studies have observed a reduction in ischemia-reperfusion injury (IRI) due to MP [14, 15].

A well-studied modality of MP is end-ischemic hypothermic oxygenated MP. It is a relatively simple modality of MP which is performed for a short period of two hours after conventional SCS during which the liver is transported to the hospital of the recipient [15, 16]. Experimental animal studies have demonstrated improved cellular energy homeostasis due to end-ischemic oxygenated MP by restoration of mitochondrial function which resulted in an increased adenosine triphosphate (ATP) tissue concentration. Other observed effects are reduced production of reactive oxygen species, reduced cellular death [14–16], improved hepatocyte function, and enhanced energy dependent bile production after warm reperfusion [17]. Furthermore, the injury to the vascular endothelium [15] and peribiliary vascular plexus [18] is attenuated. To summarize, experimental animal studies have shown that end-ischemic hypothermic oxygenated MP of liver grafts after SCS reduces IRI resulting in improved organ integrity and function after reperfusion.

Based on the excellent results in experimental studies, end-ischemic hypothermic MP was investigated in the clinical setting of human liver transplantation in hospitals in New York, Zurich, and Groningen. These first clinical experiences have shown that the preservation method is safe, improves early graft function, and attenuates IRI as reflected by a reduction of postoperative serum markers of liver preservation injury. Furthermore, fewer complications such as NAS and shorter hospital stay were observed in comparison to a retrospective control group of patients receiving a liver preserved with SCS alone [19–22].

Although the results of the first clinical studies are promising, they were small cohort studies without a randomized control group. The present study is a randomized controlled trial to determine the efficacy of two hours of end-ischemic DHOPE prior to implantation of a DCD (Maastricht category III) liver graft in reducing the incidence of NAS after transplantation.

## Methods

### Design and objective

The DHOPE-DCD trial (dual hypothermic oxygenated machine perfusion in donation after circulatory death liver transplantation) is designed as a prospective, randomized, controlled, multicenter, parallel-arm, inferiority, clinical trial in 156 patients undergoing DCD liver transplantation. The primary objective is to study the efficacy of end-ischemic DHOPE in reducing the incidence of symptomatic NAS after DCD liver transplantation. Liver grafts in the intervention group will be preserved with SCS followed by two hours of DHOPE and liver grafts in the control group will be preserved by SCS alone without any further intervention. This multicenter trial is investigator-initiated and at least five academic centers are included, located in the Netherlands, Belgium, and the United Kingdom. A list of study sites can be obtained via Clinicaltrials.gov with the identifier NCT02584283 (https://clinicaltrials.gov/ct2/show/NCT02584283) where the trial was registered in September 2015 and periodically updated. This article concerns study protocol version 4.0, 6 September, 2018.

### Study endpoints

The primary endpoint is the incidence of symptomatic NAS at six months after DCD liver transplantation. The diagnosis of symptomatic NAS is defined according to all of the following criteria [23]:

- clinical signs (i.e., jaundice, cholangitis) or elevation of cholestatic laboratory parameters in blood samples taken during follow-up
- any irregularities or narrowing of the lumen of the intra- or extrahepatic donor bile ducts, (isolated strictures at the bile duct anastomosis were not defined as NAS)
- which are diagnosed by cholangiogram (preferably by MRCP)
- in the presence of a patent hepatic artery demonstrated by Doppler ultrasonography and if necessary, by computed tomography angiography
- and as assessed by the Adjudication Committee

The secondary endpoints (summarized in Table 1) are the following:

1. The overall incidence of NAS including both symptomatic and asymptomatic NAS. Asymptomatic NAS is defined according to all of the criteria for symptomatic NAS but excludes clinical signs (i.e., jaundice, cholangitis) or elevation of cholestatic laboratory parameters in blood samples taken during follow-up. Patients will undergo an MRCP at six months after transplantation (time window of 15 days), unless they have been diagnosed with NAS within 6 months after transplantation or have been retransplanted.
2. The severity and location of NAS based on:
  - Scoring system described by Buis et al. [23]
  - Required treatment for NAS (i.e. ursodeoxycholic acid, endoscopic stenting, or retransplantation)
3. Graft (censored and uncensored for patient death) and patient survival at 7 days, 1, 3, and 6 months
4. Primary non-function defined as liver failure requiring retransplantation or leading to death within seven days after transplantation without any identifiable cause such as surgical problems, hepatic artery thrombosis, portal vein thrombosis, and acute rejection [24].
5. Initial poor function which is based on a modification of the Olthoff criteria: international normalized ratio (INR) > 1.6 and/or serum total bilirubin > 10 mg/dL on postoperative day 7 [25]. If there are multiple analyses in one day, the morning sample at around 5.00 A.M. is registered.
6. Graft function and ischemia-reperfusion injury determined by serum levels of alanine aminotransferase (ALT), aspartate aminotransferase (AST), alkaline phosphatase (AlkP), gamma-glutamyl transferase (γGT), and total bilirubin at postoperative day 0–7 and 1, 3, and 6 months. Day 0 is defined as the interval between graft portal reperfusion and the midnight of that day. If there are multiple analyses in one day, the morning sample at around 5.00 A.M. is registered.
7. Hemodynamic status (blood pressure, heart rate and vasopressor dosage) at 5 min before reperfusion, as well as 10 and 20 min after reperfusion
8. Length of initial ICU and initial hospital stay determined in days of admission following liver transplantation. Duration of follow-up hospital stay is determined in days of hospital admission after discharge and up to six months after liver transplantation.
9. Postoperative complications are graded according to the comprehensive complication index (CCI) [26]. Special interest will be given to predefined infectious complications and the total length of use and cumulative doses of antibiotics.
10. Renal function which is defined as
  - Estimated glomerular filtration rate (eGFR) according to the 4-variable Modification of Diet in Renal Disease (MDRD) equation [27] at day 7, and 1, 3, and 6 months after transplantation. If there are multiple analyses in one day, the morning sample at around 5.00 A.M. is registered.
  - Kidney injury is scored according to acute kidney injury network (AKIN) and risk, injury, failure, loss of kidney function and end-stage kidney disease (RIFLE) criteria [28].
  - In selected centers, urinary kidney injury markers such as kidney injury molecule (KIM) [29], tissue inhibitor of matrix metalloproteinases-2 (TIMP2), Insulin-like Growth Factor Binding Protein-7 (IGFBP7), and neutrophil gelatinase-associated lipocalin (NGAL) will be measured in urine samples taken at arrival in the ICU and at day 1, 3, and 5 after transplantation.
11. Perfusion characteristics during DHOPE include flow, pressure and resistance at every fifteen minutes.
12. In selected centers, perfusate analyses will be performed to study the dynamics of experimental markers of tissue and mitochondrial injury. The perfusate at the start and end of DHOPE procedure, and every half hour in between will be analyzed for pH, sodium, potassium, bicarbonate, lactate, ALT, AST, AlkP, γGT, urea, total bilirubin, thrombomodulin, high mobility group box-1 (HMBG) protein, and cytochrome C [30].
13. In selected centers, prognostication of NAS based on miRNA’s: CDmiR-30e, CDmiR-222, CDmiR-296, HDmiR-122 and HDmiR-148a will be determined in perfusate.
14. In selected centers, biopsies of liver parenchyma and bile duct, which are routinely taken during transplantation, are also taken in this trial. The biopsies are taken at three time points: before DHOPE, after DHOPE, and after reperfusion at the time of bile duct anastomosis during anesthesia. The purpose is to underpin the histopathological status of the liver and bile ducts in both study groups. In addition, mechanistic research into molecular mechanisms of injury and repair during DHOPE will be done to identify pathophysiological pathways that might have potential to predict function and outcomes after transplantation.
15. Metabolic function, including new onset diabetes after transplantation (NODAT) in the first 90 days after transplantation. NODAT is defined according to the WHO criteria (Report 2003 S5–20).

- Symptoms of diabetes and random plasma glucose ≥11.1 mmol/L. Symptoms include polyuria, polydipsia, and unexplained weight loss. OR
- Fasting plasma glucose ≥7.0 mmol/L. Fasting is defined as no caloric intake for at least eight hours. OR
- Two-hour plasma glucose ≥11.1 mmol/L during an oral glucose tolerance test. The test should be performed as described by the WHO, using a glucose load containing the equivalent of 75 g anhydrous glucose dissolved in water.

16. In selected centers, overall cost of treatment within 6 months (in/excluding return to work) is calculated according to the Cost and Outcome analysis of Liver Transplantation (COLT) study [5].
17. Health related quality of life will be determined using an EQ-5D-3 L questionnaire obtained before transplantation and at 6 months after transplantation. The EQ-5D-3 L is a generic questionnaire and covers five domains of health (mobility, self-care, usual activities, pain/discomfort, and anxiety/depression).

**Table 1:** Endpoints

Primary endpoint	
Symptomatic NAS	
Secondary endpoint	
1. Incidence of (a)symptomatic NAS	
2. Severity of NAS	
3. Graft and recipient survival	
4. Primary non-function	
5. Initial poor function	
6. Serum biochemical graft function and injury	
7. Hemodynamic status during reperfusion	
8. ICU and hospital stay	
9. Postoperative complications	
10. Renal function	
*DHOPE group only:*	
11. Perfusion characteristics	
12. Perfusate biochemical graft function and injury	
13. Perfusate micro ribonucleic acid (miRNA)	
14. Pathobiology of liver and bile duct parenchyma	
15. Metabolic function, including new onset diabetes after transplantation (NODAT)	
16. Costs	
17. Health related quality of life	

*Abbreviations: DHOPE* dual hypothermic oxygenated machine perfusion, *ICU* intensive care unit, *NAS* non-anastomotic biliary strictures

### Study population

Adult patients (≥ 18 years old) eligible for liver transplantation are screened for participation in this trial. The inclusion and exclusion criteria are presented in Table 2.

**Table 2:** Inclusion and exclusion criteria

Inclusion	Exclusion
Adult patients (≥ 18 years old)	Contraindication for MRCP
Donor liver from DCD (Maastricht category III)	Listed for fulminant liver failure or retransplantation due to primary non-function
Donor body weight ≥ 40 kg	Simultaneous transplantation of another organ
Signed informed consent	Incapable to give informed consent due to mental conditions
	Simultaneous participation in another clinical trial that might possibly influence this trial
	Recipient positive test for HIV antigen or antibody
	Donor positive for HIV antigen or antibody, Hepatitis B core antibody or hepatitis B surface antigen, or hepatitis C antibody

*Abbreviations: DCD* donation after circulatory death, *HIV* human immunodeficiency virus, *MRCP* magnetic resonance cholangiopancreatography

### Randomization

Randomization is performed when a DCD donor liver becomes available for a patient who is eligible for the DHOPE-DCD Trial and has given informed consent. However, the patient is only randomized when the liver is definitively deemed suitable for transplantation and the rest of the transplant team including the anesthetist is informed about the exact starting time of the transplantation. The randomization takes place via an online web-based tool and is performed by trained trial personnel. It is a block randomization which is stratified for trial site and for patients with primary sclerosing cholangitis.

### Blinding

The procurement surgeon is blinded for the study group assignment during organ retrieval. Patients are also blinded to study group assignment. Additionally, assessment of the primary endpoint is performed by the blinded Adjudication Committee. When there is a breach of blinding, this is described in the eCRF and the Sponsor is notified.

### Study procedures

**Fig. 1:**
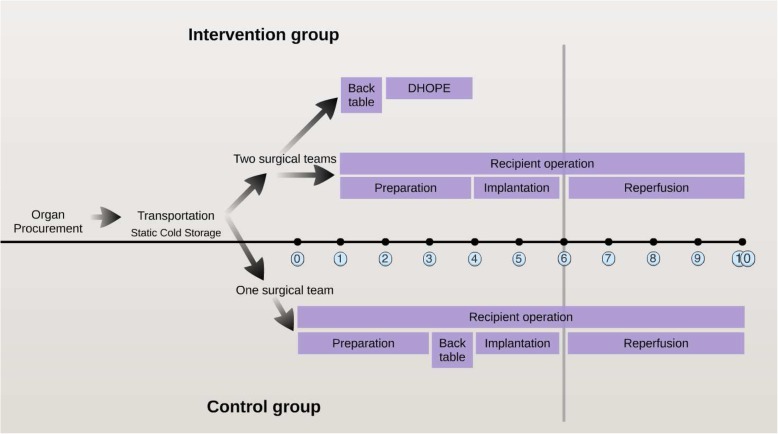
Flow chart of study procedures. Abbreviations: DHOPE, dual hypothermic oxygenated machine perfusion

The timeline of study procedures are graphically presented in Fig 1.

#### Procurement of the donor liver (both study groups)

After circulatory death of the donor, the stand-by surgical team performs a median laparotomy and aortic cannulation to perfuse the abdominal organs with at least 4000 ml of cold (0–4 °C) preservation fluid, preferably Belzer UW® Cold Storage Solution (Bridge-to-Life, Ltd., Northbrook, IL) (UW CS) with 50.000 IU of heparin. The liver is procured with a segment of 5 cm circular supratruncal aorta left attached to the coeliac trunk if possible. The portal vein and common bile duct are kept as long as possible. After procurement the liver is flushed via the portal vein with at least 1 l of preservation fluid, preferably UW CS, without applying pressure. The cystic duct is ligated and the bile ducts are flushed with preservation fluid, preferably UW CS and preferably at the donor hospital. The gallbladder is preferably left in situ. The liver is transported to the recipient hospital where the conventional back table procedure is performed.

#### Back table (DHOPE group)

During the back table procedure, the portal vein and the supratruncal aorta are cannulated. The supratruncal aorta is cannulated in a manner that the hepatic artery is not damaged due to cannulation. The side branches of the hepatic artery are ligated or clipped during the back table preparation. Short before connection to the machine, the liver is flushed via the portal vein cannula with 1000 mL cold (0–4 °C) Belzer MPS® UW machine perfusion solution (Bridge-to-Life, Ltd., Northbrook, IL) (UW MP) until the caval effluent is clear. Normally the back table preparation takes one hour on average.

#### Investigational medical device (DHOPE group)

Simultaneously with the back table procedure, the Liver Assist (LA) is prepared for use. The LA is a dedicated machine for ex-vivo liver perfusion during storage. It is a CE marked device (European Union Certification of Safety, Health and Environmental Requirements) that is designed, produced, and delivered by Organ Assist (Groningen, The Netherlands). The LA has been used in a pilot study (www.trialregister.nl, NTR4493, van Rijn et al. [31]) and is currently in use in Zurich in a randomized controlled trial in transplantation of donation after brain death (DBD) liver grafts (clinicaltrials.gov identifier NCT01317342). The LA enables dual perfusion via the portal vein and the hepatic artery using two centrifugal pumps to provide a continuous venous flow and a pulsatile arterial flow at 60 bpm. The system is pressure controlled which allows autoregulation of the flow through the liver, with constant pressure at variable flow rates. The perfusion fluid can be oxygenated by two hollow fiber membrane oxygenators and carbon dioxide can be removed. The temperature of the preservation fluid can be adjusted between 10 and 38 °C. The system can be filled with any preferred perfusion fluid.

#### Preparation of the liver assist (DHOPE group)

The LA is prepared by connecting the disposable to the machine and filling the disposable with 4000 mL ice-cold UW MP with additional 3 mmol/L glutathione (Biomedica, Foscama Group, Roma, Italy). The gluthatione is a regular component of the Belzer MPS® UW machine perfusion solution. As gluthatione can become inactive during shelf-time, the manufacturer recommends addition before use. The perfusion pressure will be limited to a mean of 25 mmHg for the hepatic artery and 5 mmHg for the portal vein. These pressure settings are based on previous studies and are lower than physiological pressures to avoid shear stress of the cold endothelium of the hepatic vasculature [15, 18, 32]. The temperature of the perfusion fluid will be about 12 °C when the temperature is set to 10 °C. The thermo-unit of the LA is filled with crushed ice in the reservoir to maintain the desired temperature. The oxygen flow is set at 500 mL/min of 100% oxygen on each of the two membrane oxygenators. This flow is adequate to obtain a pO2 which has been reported to be effective in increasing ATP and not harmful to the graft [13, 33]. The LA is ready for perfusion once the set temperature is reached and the solution is oxygenated for at least 15 min with oxygen.

#### Perfusion of liver (DHOPE group)

The surgeon connects the cannulas to the disposable tubing of the LA after which the pumps of the device are started. During the first five minutes after the connection of the liver to the machine, the perfusion pressure will be increased with 2 mmHg if the flow is less than 100 ml/min flow. To maintain the temperature, the crushed ice in the reservoir of the thermo-unit must be replaced regularly during perfusion. A surgeon will supervise the perfusion and will be available for trouble shooting.

#### Follow-up (both study groups)

During follow-up serum laboratory values which are routinely assessed are monitored (Table 3). At 6 months after transplantation, the patients will undergo an MRCP and fill in the questionnaire on health related quality of life (EQ-5D-3 L). The MRCP will be planned during a routine hospital visit to the out-patient department for a regular check-up. The MRCP will be cancelled in case the patient has undergone retransplantation or has been diagnosed with symptomatic NAS before 6 months of follow-up.

**Table 3:** Laboratory parameters

	Donor	Recipient	
		Baseline	Follow-up
*Parameter*	*Latest before procurement*	*Latest before transplantation*	*Day 0–7, Month 1,3,6*
Alanine aminotransferase (ALT)	X		X
Aspartate aminotransferase (AST)	X		X
Alkaline phosphatase (AlkP)	X		X
Gamma-glutamyl transferase (γGT)	X		X
Total bilirubin	X	X	X
Lactate dehydrogenase (LDH)	X		X
Creatinine	X	X	X
Potassium	X		X
Sodium	X		
International normalized ratio (INR)		X	X
Thrombocytes			X
Glucose			X

Day 0 is determined as the interval between graft portal reperfusion and the midnight of that day. If there are multiple analyses in one day, the morning sample at around 5.00 A.M. is registered

### Usual care

Patients assigned to the control group will be transplanted with a liver graft preserved with SCS. The patients will receive the routine health care given at participating centers. Diagnostic and treatment decisions will not be influenced by the trial procedures.

### Data and material collection

Study data will be anonymously registered under a unique study number in an electronic case report form (eCRF) via a web-based tool. Patient’s name, address and date of birth will be stored separately from the trial data. The investigators, members of the Health Inspection and members of the Medical Ethical Committee (MEC) will have access to personal data. Study data and human material will be stored during 15 years. We expect that the stored material will be valuable for future research. Assess to the final trial data set will be coordinated by the Sponsor.

### Statistical analysis

#### Power calculation

The study is powered to detect a clinically relevant difference in incidence of NAS between the two study groups. The incidence of NAS was 29% after DCD liver transplantation and 11% after DBD liver transplantation in patients transplanted in the UMCG from 2008 to 2013 (unpublished data). This incidence is similar to that reported by Abt et al. (27% in DCD versus 2% in DBD transplantation), Dubbeld et al. (24% versus 8%), Croome et al. (22% versus 4%), and Meurisse et al. (33% versus 12%) [6, 34–36]. We aim to reduce the incidence of NAS after DCD liver transplantation with DHOPE to the level observed after DBD liver transplantation (absolute difference of 29–11 = 18%). We base this presumed reduction on our results in the pilot study in which 1 of 10 (10%) of the patients with a DHOPE treated liver developed NAS. Moreover, in other phase-1 studies of hypothermic machine perfusion no patients developed NAS [19–22]. Based on a power of 80% (β = 0.80) and a 5% significance level (2-sided test) in two independent cohorts, a Chi-squared test indicated that 77 livers were needed in each study arm (nQuery + nInterim 3.0). Although there is a (very) small likelihood of lost to follow-up, we still want to include an extra patient per study arm. In conclusion, the total number of patients to be included in this study will be 156 (77*2 + 2).

### Trial sites

Participating sites are:

1. University Medical Center Groningen, Groningen, the Netherlands
2. Erasmus University Medical Center, Rotterdam, the Netherlands
3. Leiden University Medical Center, Leiden, the Netherlands
4. University Hospitals Leuven, Leuven, Belgium
5. Ghent University Hospital, Ghent, Belgium
6. King’s College Hospital NHS Foundation Trust, London, the United Kingdom
7. Queen Elizabeth Hospital Birmingham, University Hospitals Birmingham NHS Foundation Trust, Birmingham, the United Kingdom

### Monitoring

The monitoring will be performed by qualified monitors associated with the Trial Coordinating Center of the UMCG or of the participating Trial Site. They will periodically review whether study procedures are performed in accordance with Good Clinical Practice (GCP) and the study protocol with regard to informed consent, randomization, study end points, reporting of adverse events, etc. They will advise the Sponsor if, in its view, the study should be terminated due to major deviations from the study protocol.

### Safety monitoring

In accordance to section 10, subsection 1, of the Wet medisch-wetenschappelijk onderzoek (English: Law Medical Research), the Investigator will inform the subjects and the reviewing accredited MEC if anything occurs, on the basis of which it appears that the disadvantages of participation may be significantly greater than was foreseen in the research proposal. The trial will be suspended pending further review by the accredited MEC, except insofar as suspension would jeopardize the subjects’ health. The Investigator will take care that all subjects are kept informed.

#### Data safety monitoring board (DSMB)

To ensure the safeguarding of the included patients and the expected additional burden with trial participation, a DSMB with independent experts has been installed. The advice(s) of the DSMB will be provided on a regular basis after receipt, review and analysis of the interim and final efficacy and safety data to the Sponsor and Investigators and to the MEC that approved the protocol.

An interim analysis will be performed by the DSMB after half of all patients (156/2 = 78) are included in the trial and have completed follow-up of 6 months. The interim analysis aims to determine the incidence of NAS in the control group as a means of determining whether the assumption in our sample size calculation was correct: 29% patients develop NAS after DCD liver transplantation.

No statistical test will be performed unless concerns arise about the safety of DHOPE based on the incidence of adverse events. In that case, the Pocock sequential boundary will be used to determine statistical significance of adverse events between the two groups, dictating a Z-value of 2 and thus a *P*-value of 0.045. The trial may be stopped early due to: unacceptable safety concerns with significantly more (serious) adverse events in the intervention group compared to the control group and in case new external information arises that convincingly answers the study question or raises serious safety issues.

#### Reporting

All adverse events will be recorded in the eCRF and reported in line listing to the MEC and the DSMB every six months. Serious adverse events (Table 4) and suspected unexpected serious adverse reactions (SUSAR) will be reported real time to the Sponsor, the MEC, and the DSMB. Since liver transplantation is a surgical procedure with significant morbidity, frequently occurring complications will be exempt from real time reporting, but will be reported only in the eCRF and every six months in line listing.

**Table 4:** Serious adverse events

Serious adverse events	
Death	
Graft failure (or retransplantation)	
Primary non-function	
Vascular complications (e.g. thrombosis) of	
Portal vein	
Hepatic artery	
Device deficiency	
Malfunction	
Use errors	
Which lead or could have led to serious adverse events	
Unanticipated serious adverse device effects (USADEs)	

### Ethical consideration and informed consent

This trial will be conducted in accordance with the principles of the Declaration of Helsinki and as stated in the laws governing human research in the participating countries and Good Clinical Practice. The MEC of the University Medical Center Groningen and local ethical committees of all participating centers have approved this protocol. This protocol was endorsed by the board of directors of each participating hospital prior to the inclusion of subjects at the respective hospital.

It is important to note that absolutely no changes will be made to national and international liver allocation rules. The standard local liver allocation rules will be followed. The study does not interfere or change the process of accepting or declining a liver offered to a certain patient in any way.

#### Withdrawal of treatment

Subjects and Investigator can decide to terminate participation in this trial at any time for any reason, particular safety reasons, if they wish to do so without any consequences.

#### Informed consent

Informed consent will be obtained by trained trial personnel from each participating patient in oral and written form prior to randomization. We chose to ask eligible patients for informed consent while they are on the waiting list or when they are placed instead of obtaining consent when the patient is allocated a DCD liver. By choosing this method of informed consent, many patients will have given informed consent but will not be included in the study because they will receive a liver from a donation after brain death donor instead of a DCD liver. However, we believe that it is less stressful for patients to give consent in a stable situation when they are listed for liver transplantation (either DCD or DBD) at the outpatient department than when they are summoned to the hospital for a DCD liver transplantation. Therefore, the patient will be merely informed whether they are included in the trial at the time they are summoned to the hospital for the transplantation.

### Additional burden and risk associated with trial participation

The potential risk and burden to the patients in this trial are minimal and are overshadowed by the potential benefit if our hypothesis is correct: DHOPE reduces the risk of patients developing NAS after liver transplantation. NAS are of major impact to the patient since they often imply a multitude of costly and debilitating interventions and hospital admissions which may be unsuccessful and eventually require retransplantation. Additionally, experience with DHOPE in clinical liver transplantations indicate decreased preservation injury, improved organ integrity and function, and experience with experimental animal models showed increased survival after liver transplantation in the DHOPE preserved livers.

The minimal potential risk and burden for patients in this trial are related to the three procedures which are performed merely for this trial: DHOPE itself, the MRCP and the questionnaire about health related quality of life. Although we regard the risk assessment of DHOPE as minimal, the risk could be associated with the timing, the perfusion fluid, the pressure used, and the device. DHOPE is technically a relatively easy procedure which can be performed by any liver surgeon after training. As perfusion is performed in the transplantation center while the recipient undergoes surgery, there is no alteration in transportation of the organ by means of conventional SCS.

The two hours of DHOPE performed at the end of SCS will cause a minor delay in the transplantation process because the preparation of the liver for transplantation and the DHOPE perfusion will be performed simultaneously with the recipient operation and will be performed by an extra surgical team (Fig. 1). Normally, preparation of the liver is performed by the surgeons who perform the transplant operation, but this can be performed simultaneously by another team during this trial.

Furthermore, DHOPE is performed using a machine, which could display technical failure during perfusion. If a technical failure occurs, an alarm goes off and the liver can be removed immediately from the machine by standby surgeon within a sterile environment. It will be cooled down in preservation fluid from 12 °C (in DHOPE) to 4 °C as is the case in SCS. For some minutes before the liver is cooled down, it has a metabolism of 19% instead of 11% as is the case in SCS. There is no reason to believe that this event will cause any significant injury to the liver grafts because this difference in metabolism is very small, is of short duration, and the liver is saturated with oxygen before the perfusion failure. If the malfunctioning LA cannot be fixed, the donor liver will be preserved in SCS until implantation and analysis of the study results will be performed as an intention-to-treat analysis: the patient will remain in the intervention group.

Moreover the preservation fluid used is similar to the one used in the control group. The only difference is that the sodium and the potassium concentration ratio are inverted. Therefore, the components that might cause an allergic reaction in the patient receiving the liver are no different from standard practice.

Lastly, the perfusion pressure could theoretically harm the organ. However, the pressures used in this trial are very low (lower than physiological) and are reported to be used safely without causing any harm to the organ or the vasculature [32, 37].

In the unfortunate event in which injury or death is caused by the study, the trial centers have an insurance policy which is in accordance with the legal requirements and provides coverage for damage to trial participants.

#### Risks and burden associated with MRCP

The MRCP will be performed at six months after transplantation in patients with no history of NAS or graft loss. The risks associated with this imaging modality are insignificant. It is a non-invasive test taking about 45 min. The MRCP will be planned on the same day that the patient has a routine 6-month check-up in the outpatient department to minimize patient’s traveling.

#### Burden associated with questionnaire

The questionnaire on health related quality of life consists of 6 questions and is completed in about 5 min. Since the questionnaire is completed twice (before transplantation and at 6 months after transplantation), the burden of the questionnaire is very low.

## Discussion

DCD liver grafts are increasingly used for transplantation in an attempt to overcome the organ shortage. In the USA, transplantation of DCD livers accounted for 6% of all liver transplantations performed in 2013, whereas in the UK and The Netherlands as much as 33% of liver transplantations in 2015 were performed with a DCD liver [3, 4, 38]. The major drawback of DCD, compared to DBD, is the inevitable period of warm ischemia which leads to the depletion of intracellular energy sources, such as ATP as well as other metabolic perturbations causing cellular injury and dysfunction [39, 40]. This damage is exacerbated by reperfusion injury of the liver graft and is clinically manifested as an increased risk of complications and graft failure after transplantation [34]. The most troublesome complications after DCD liver transplantation are biliary complications such as NAS, which have been reported in up to 30% of the patients after DCD liver transplantation compared to 10% in recipients of a DBD liver graft [36, 41, 42].

This randomized controlled trial aims to study the efficacy of a new preservation method, end-ischemic DHOPE, in reducing the incidence of NAS after DCD liver transplantation. End-ischemic hypothermic machine perfusion has been studied previously in phase-1 non-randomized trials in human orthotopic liver transplantation [19–22]. These first clinical experiences have provided promising results such as improved early graft function, attenuated classical biochemical markers of liver preservation injury, fewer (biliary) complications, and shorter hospital stay in comparison with a retrospective control group of patients receiving a liver preserved with SCS alone.

The rationale of this study is based on the results of the abovementioned phase-1 trials [11, 12, 16, 17]. However, these first clinical trials were cohort studies with retrospectively selected non-randomized control groups. Therefore, it is unwise and perhaps even unethical to adjust usual care as long as the clinical and health economic benefit of DHOPE has not been demonstrated in a randomized controlled trial.

The primary endpoint of this study is symptomatic NAS within six months after transplantation. This clinical endpoint is selected as it is considered to reflect a relevant sign of biliary injury caused by ischemia-reperfusion injury [8, 23]. Also, diagnostic imaging is reproducibly attainable at all study sites and therefore can be objectified by blinded assessment by the Adjudication Committee including independent radiologists. Moreover, the preferred imaging modality is MRCP which is minimally invasive and is part of the routine diagnostic work-up in case of clinical suspicion of NAS. For the primary endpoint, the time interval of six months after transplantation is chosen because the diagnosis of NAS is reported at a median of three to four months after transplantation [23, 43]. Some studies have reported an occurrence of 100% of the cases of NAS within four months [33, 43–45]. Furthermore, when NAS does develop more than 6 months following transplantation, it is less likely mediated by ischemia-reperfusion injury [8, 46].

According to the study protocol, all patients undergo an MRCP at 6 months after transplantation regardless whether they have symptoms of cholestasis. This MRCP will be assessed for biliary strictures by the blinded Adjudication Committee. With this standardized MRCP, the risk of a potential bias in diagnosis rate of NAS will be minimalized, for example when physicians would deliberately postpone the diagnostic imaging for NAS until after 6 months of follow-up. Conversely, in patients with graft failure or NAS before 6 months of follow-up, the planned MRCP at 6 months will be cancelled.

A potential pitfall of the study is the risk of declining the DCD liver for transplantation after randomization. To overcome a bias due to a physicians’ preference for either study group, the protocol stipulates randomization after the definitive accept of the liver for transplantation.. Additionally, to ensure identical procurement of the donor liver, the surgeons performing the procurement will be blinded for the study group assignment during the organ recovery procedure.

Inclusion criteria are chosen to optimally reflect the current clinical practice. Selective inclusion would certainly hamper the future implementation of results. For this reason, patients with a retransplantation using a DCD liver are included in this study. Pre-selection by excluding patients with retransplantation would potentially optimize patient outcome, but not reflect the current clinical practice in which organ scarcity forces clinicians to weigh waiting list time against graft quality. Nevertheless, patients with fulminant liver failure are excluded from this trial as their high risk of complications and mortality would potentially weaken safety monitoring of the trial. Also, donors with HIV, hepatitis B, or C are excluded from participating in order to minimize the risk of contamination of the medical device. A similar approach is applied in renal replacement therapy where patients with HIV or hepatitis B or C are dialyzed with dedicated machines to avoid contamination of non-infected patients.

End-ischemic hypothermic machine perfusion is a rapidly developing and dynamic field with yet many still unanswered questions. For example, there is no consensus on the need for active oxygenation or single (portal vein) versus dual perfusion (hepatic artery and portal vein). Guarrera et al. were the first to report successful clinical transplantation of extended criteria DBD donor livers after ex situ hypothermic machine perfusion (4–6 °C) via the portal vein and hepatic artery without active oxygenation [19, 20]. Dutkowski et al. subsequently reported that active oxygenation of the perfusion fluid adds significantly to the benefits of hypothermic (10 °C) perfusion in DCD liver transplantation [15, 19, 20, 47, 48]. Although Dutkowski’s group applied active oxygenation of the perfusion fluid, they only perfused via the portal vein and not via the hepatic artery. Up to present it remains unclear whether dual or single perfusion is equally effective or one method is superior to the other. However, as biliary complications are the main obstacle for wider utilization of DCD livers and the bile ducts are known to be predominantly vascularized through the artery, the single portal perfusion may (at least in theory) not provide optimal preservation of the bile ducts and their vasculature [49].

In conclusion, the DHOPE-DCD trial is a multicenter trial designed to assess the effect of dual hypothermic oxygenated machine perfusion compared to SCS on the incidence of non-anastomotic biliary strictures in DCD liver transplantation. This trial aims to improve the outcome of patients transplanted with a DCD donor liver by reducing the risk of NAS. Therefore, the DHOPE-DCD trial has the potential to impact the outcome of the individual patient by decreasing the risk of and retransplantation. Secondly, this trial may impact the outcome of all patients awaiting a liver transplantation because a decrease in retransplantation rate would lead to more remaining available liver grafts for other patients. Finally, this study examines the cost-effectiveness and budgetary impact of DHOPE against usual care for patients with DCD liver transplantation and may affect future health care policy concerning DCD liver transplantation.

## Abbreviations

AKIN: Acute kidney injury network
AlkP: Alkaline phosphatase
ALT: Alanine aminotransferase
AST: Aspartate aminotransferase
ATP: Adenosine triphosphate
CCI: Comprehensive complication index
COLT: Cost and outcome analysis of liver transplantation
DBD: Donation after brain death
DCD: Donation after circulatory death
DHOPE: Dual hypothermic oxygenated machine perfusion
DSMB: Data safety monitoring board
eCRF: Electronic case report form
eGFR: Estimated glomerular filtration rate
GCP: Good clinical practice
HMBG-1: High mobility group box-1
ICU: Intensive care unit
IGFBP7: Insulin-like growth factor binding protein-7
INR: International normalized ratio
IRI: Ischemia reperfusion injury
MDRD: Modification of diet in renal disease
MEC: Medical ethical committee
MP: Machine perfusion
MRCP: Magnetic resonance cholangiopancreatography
NAS: Non-anastomotic biliary strictures
NGAL: Neutrophil gelatinase-associated lipocalin
NODAT: New onset diabetes after transplantation
RIFLE: Risk, injury, failure, loss of kidney function and end-stage kidney disease
SCS: Static cold storage
SUSAR: Suspected unexpected serious adverse reactions
TIMP2: Tissue inhibitor of matrix metalloproteinases-2
UW CS: University of Wisconsin cold storage solution
UW MP: University of Wisconsin machine perfusion solution
γGT: Gamma-glutamyl transferase
